# The National Veterinary Association of Great Britain

**Published:** 1886-10

**Authors:** 


### THE NATIONAL VETERINARY ASSOCIATION OF GREAT BRITAIN.

The fourth annual meeting of the National Veterinary Association opened in Edinburgh, July 22, 1886. Principal Walley, of the Royal (Dick) Veterinary College, Edinburgh, presiding, and Mr. George A. Banham, Cambridge, as secretary.

The President, in his opening address, welcomed the Association to Edinburgh, which to all veterinary surgeons, he said, was classical ground, for here, upwards of a century ago, the pioneer and father of veterinary surgery was born, and upwards of seventy years ago he commenced, under disadvantageous cirumstances, to teach the practice of veterinary medicine. It was a fortunate day for the profession when Professor Dick turned his back on the London Veterinary College, and sturdily retraced his steps to Scotland and established a veterinary school of his own. Having referred to the fact that Edinburgh was the only city in the United Kingdom that was able to support more than one veterinary school, Principal Walley made a kindly allusion to the recognition given to their profession by the Highland and Agricultural Society for sixty years. After sketching the origin and progress of the National Veterinary Association, and the objects it had in view, the President briefly remarked on the immense strides made in the unification and consolidation of the profession during the last twenty years, and, in conclusion, expressed the hope that the day was not far distant when the Government would recognize the importance of the veterinary profession in the economy of the country by extending to it a helping hand. On the motion of Principal Williams—who referred to the valuable services to scientific thought of the late John Barlow—a hearty vote of thanks was given to Principal Walley for his address.

The first paper taken up for discussion was one by Mr. J. S. Hurndall, on the question—"Can experimental pathogenesy be rendered useful in elucidating a definite system of veterinary therapeutics?" The writer asserted that their therapeutics were not up to the present day practice, and called aloud for revision. The teaching of therapeutics in the schools was inadequate to fit their members for their duties, if they were to have an enlightened scientific method of administering drugs, and not a bald, empirical method. He proceeded to refer to some diseases or morbid conditions which caused the practitioner considerable anxiety, and he argued from the methods of treatment that there was urgent necessity for a revision of their therapeutics. He took as an illustration bronchitis, and recommended the administration of aconite and bryonia. Pathogenetic experimentation on animals in health was, he said, not only reasonable, but likely to prove of the highest value to the veterinary profession, provided they were carried out systematically and with the closest observation.

Mr. T. Hopkin combated the views of the essayist, and demurred to his recommendation that they should rely so largely on drugs. Without the *vis naturæ* they might throw physic to the dogs. Principal Williams said the paper had a high-sounding title, but it really amounted to nothing but homœopathy. The principle of treating disease with the view of combating the symptoms was irrational. Physiologically, aconite had the effect of soothing pain and reducing the action of the heart, but it had no specific effect on the bronchial tubes. It gave the animal repose, and they all knew that rest was the greatest factor in the cure of disease. But because they should follow the dictates of nature in the treatment of disease, he did not support the notion that disease ought to be allowed to take its course without any remedy being attempted. It was by following the dictates of nature, by the scientific application of remedies when required, by a reliance on the *vis medicatrix naturæ*, and by abandoning the "heroic measures" that appertained in his younger days, that they would become successful in their treatment. Dr. Fleming, said the better they understood physiological processes the better could they carry out pathological treatment. As a veterinary surgeon of over twenty-five years experience, he seldom gave a dose of medicine, and he had been as successful as other practitioners. He impressed upon young members of the profession that they should give less medicine, and attend more to the requirements of nature. Principal Walley said he would administer aconite in certain circumstances, but not in those recommended by the essayist. He knew from experience that they could give aconite with bicarbonate of potash or other alkaloid, providing they gave it in ball form, but if administered as a draught the chances were they would kill their subject. Mr. Hurndall replying to the discussion, said he did not understand the power of nature, but contended that nature was incapable of doing all that was required. Relying upon nature unaided was like trusting to a broken reed. He agreed that they could not cure disease, but could only assist nature, and that was what he aimed at. He regretted the introduction of the term "homœopathy." His object had been, not to sustain the principles of homœopathy, but to convince them that investigation into the action of drugs was an essential at this time. He admitted that the principle he advocated was adopted by homœopathists, but that was an entirely different matter. His practice might be a limited one, but he invariably trusted to aconite in cases of colic, and he had yet to lose his first case.

The next subject taken up was "Notes on some of the micro-parasites of the domesticated animals," by Professor M'Fadyean, of the Royal Veterinary College, and Dr. Sims Woodhead, Pathologist to the Royal Infirmary, in which they treated of splenic apoplexy, splenic fever, anthrax, quarter-evil, black-leg, glanders, swine fever, tubercle in the udders of cattle and in milk, and louping-ill. Having narrated the symptoms of the various diseases, and given a resume of the investigations by scientists, the essayists indicate the conclusions at which they have arrived. With regard to anthrax, they are of opinion–(1) That Pasteur has succeeded in preparing a vaccin by the employment of which the domestic ruminants are put in possession of a high degree of immunity against spontaneous or inoculated anthrax; (2) that by no known method of attenuation can there be obtained a vaccin of absolutely uniform strength; (3) that it is not possible to obtain a vaccin that is at once and equally applicable to all the different species of domestic animals, or even to all the different breeds of the same species; and (4) that, even in the most capable hands, accidents capable of entailing serious results may happen in the preparation of vaccin, or in its employment. Dealing with louping-ill in sheep, the writers combat at length the views of Principal Williams in which he claimed to have proved that the disease was of a micro-parasitic nature, being caused by a bacillus.

The next paper taken up was one by Professor W. O. Williams and Mr. R. Roberts on "Anæsthetics and Anæsthesia in relation to veterinary practice." Chloroform is the best general anæsthetic. For the horse, from one and a half to two ounces usually suffices to produce insensibility for a sufficient length of time to perform a short operation. For the cow, about two ounces is required; and for the dog, about one ounce. We find that to produce a short and perfect anæsthesia the less air admitted the better. In fact, we not only cause chloroform anæsthesia, but also carbonic acid anæsthesia. It takes from five to ten minutes to produce this condition, and with this method there is little or no excitement, but when the chloroform is administered with a large quantity of air there is almost always great excitement. If the animal does not recover from the narcosis in from ten to twenty minutes after the inhalation has been stopped, we apply cold water to the head and give inhalations of ammonia. Neither of us have had a single death, and we have both performed very serious and long operations. Mr. Roberts administers anæsthetics in castration, parturition in mares, cows and bitches, removal of tumors, colic and intestinal pains, and also to cause the painless death of animals.

Mr. Owen Williams administers anæsthetics in castration of cryptorchids, removal of tumors, extraction of teeth in dogs and cats, and in causing painless death in old or useless animals, and in all serious operations. As a local anæsthetic we used cocaine, varying in quantity according to the affection, and paint it on to the parts at intervals of five minutes for half an hour before operating. Mr. Owen Williams has used cocaine in painful eye affections, has relieved the pain, and been thus enabled to apply other drugs to the parts without irritating the patient; he has also used it for the removal of vaginal tumors in the bitch, and in one case the animal watched the operations without either being tied or muzzled, and showed no symptoms of pain. We do not think that the administration of chloroform or cocaine interferes at all with the healing of the surgical wounds. Principal Williams expressed the hope that they would follow the example of the medical profession, and administer anæsthetics in all possible cases where pain would be caused by the operation.

